# An examination of multivariable Mendelian randomization in the single-sample and two-sample summary data settings

**DOI:** 10.1093/ije/dyy262

**Published:** 2018-12-10

**Authors:** Eleanor Sanderson, George Davey Smith, Frank Windmeijer, Jack Bowden

**Affiliations:** 1 MRC Integrative Epidemiology Unit, University of Bristol, Bristol, UK; 2 Population Health Sciences, University of Bristol, Bristol, UK; 3 Department of Economics, University of Bristol, Bristol, UK

**Keywords:** Mendelian randomization, two-sample Mendelian randomization, multivariable Mendelian randomization, Cochran’s Q statistic, instrument strength, instrument validity

## Abstract

**Background:**

Mendelian randomization (MR) is a powerful tool in epidemiology that can be used to estimate the causal effect of an exposure on an outcome in the presence of unobserved confounding, by utilizing genetic variants that are instrumental variables (IVs) for the exposure. This has been extended to multivariable MR (MVMR) to estimate the effect of two or more exposures on an outcome.

**Methods and results:**

We use simulations and theory to clarify the interpretation of estimated effects in a MVMR analysis under a range of underlying scenarios, where a secondary exposure acts variously as a confounder, a mediator, a pleiotropic pathway and a collider. We then describe how instrument strength and validity can be assessed for an MVMR analysis in the single-sample setting, and develop tests to assess these assumptions in the popular two-sample summary data setting. We illustrate our methods using data from UK Biobank to estimate the effect of education and cognitive ability on body mass index.

**Conclusion:**

MVMR analysis consistently estimates the direct causal effect of an exposure, or exposures, of interest and provides a powerful tool for determining causal effects in a wide range of scenarios with either individual- or summary-level data.

**Key Messages**

- Multivariable Mendelian randomization (MVMR) has been introduced as a technique for estimating the causal effect of multiple exposure variables on a health outcome with two-sample summary data. We build on this work by clarifying how MVMR should be applied with individual-level data and two-sample summary data, in order to conform with established econometric theory for multivariable two-stage least-squares analysis.
- Instrument strength and validity should be assessed in the single-sample MVMR setting using the Sanderson–Windmeijer F-statistic and the Sargan test.
- We develop a generalized version of Cochran’s Q statistic to test for instrument strength and validity in the two-sample summary data setting. However, these tests require knowledge of the covariance between the effects of the genetic variants on each exposure.
- If the covariance between the effect of the genetic variants on each exposure can be either: (i) estimated from individual data, (ii) assumed to be zero or (ii) fixed at zero by using non-overlapping samples for each exposure genome-wide association study (GWAS), then our proposed summary data Q statistics will give a good approximation of the true (individual-level data) result.
- The causal effect estimated by Mendelian randomization (MR) and MVMR can differ. MR estimates the *total* causal effect of the exposure on the outcome, whereas MVMR estimates the *direct causal* effect of each exposure on the outcome.

## Introduction

**Figure 1.**
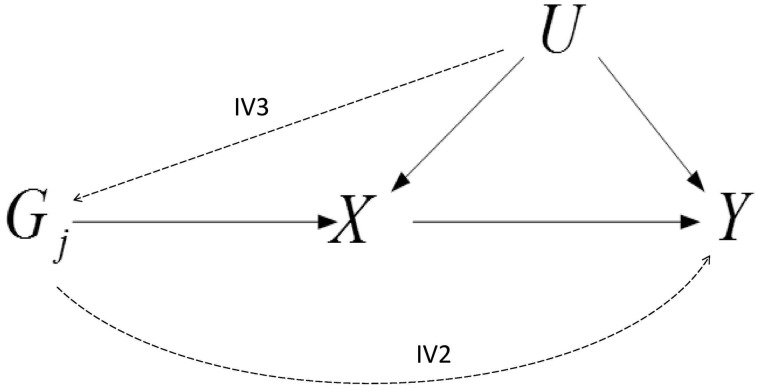
Hypothesized relationship between genetic variant(s) *G*, modifiable exposures, *X*_1_, *X*_2_ and outcome *Y* in the presence of unobserved confounder *U*. Bi-directional arrows represent possible violations of the IV assumptions induced by *X*_2_ that are explored in this paper.

In many scenarios in which we wish to estimate the causal effect of an exposure *X* on an outcome *Y*, a conventional regression analysis can be misleading, as the observational association between the two variables could easily be affected by unobserved confounding. If genetic variants—usually single-nucleotide polymorphisms (SNPs)—are available that reliably predict the exposure variable but do not have an effect on the outcome through any other pathway, then they are valid instrumental variables (IVs) and can be used in a Mendelian randomization (MR) analysis to obtain unconfounded estimates of the effect of the exposure on the outcome, as illustrated in Figure 1.

In many scenarios, we may wish to estimate the effect of multiple exposures on the outcome using MR analysis, e.g. because we believe these exposures to be closely related or because we believe one exposure may mediate the relationship between the exposure of primary interest and the outcome. This can be done with multivariable MR (MVMR) in which a set of genetic variants is used to predict a set of exposure variables. However, careful consideration needs to be given in such an analysis to exactly what relationship is being estimated and how the IV assumptions required for MR analysis apply to a MVMR analysis. In this paper, we build on previous work developing MVMR methods with two-sample summary-level data^1^^,^^2^ and fully explain how MVMR can be implemented with either individual-level or two-sample summary-level data, exactly what is being estimated in a MVMR analysis and how the IV assumptions required for MR analysis translate to MVMR analysis. We describe existing tests that can be used to test the IV assumptions with individual-level data and add to the previous literature on MVMR by developing new methods to identify potential violations of the IV assumptions with two-sample summary-level data.

### MR

To state the IV assumptions more formally with reference to Figure 1, for a single SNP $G_{j}$ to be a valid IV, it must be:

IV1: associated with the exposure X (the ‘relevance’ assumption);

IV2: independent of the outcome Y given the exposure X (the ‘exclusion restriction’); and

IV3: independent of all (observed or unobserved) confounders of X and Y, as represented by U (the ‘exchangeability’ assumption);

If IV1–IV3 are satisfied for a set of SNPs $G\;=\;\left(G_{1},\ldots ,G_{L}\right)$, then traditional IV methods can be employed to reliably test for a causal effect of X on Y using *G*, X and Y alone, without any attempt to adjust for U at all. For example, suppose the variables G, X, U and Y are linked via the following models:

(1) Y = β0 + βX + U + vy 

(2) X = π0 + πG + U + vx. 

Here, $v_{x}$ and $v_{y}$ represent independent error terms, π represents the parameter vector${\;\pi }_{1},\ldots ,\pi_{L}$ and β is the true causal effect of X on Y we wish to estimate. We will assume throughout this paper that $\left(G_{1},\ldots ,G_{L}\right)\;$are mutually uncorrelated (by design). A naïve regression of Y on X will not yield a consistent estimate for β because the explanatory variable in the regression, X, is correlated with U. However, two-stage least-squares (TSLS) estimation, performed by regressing Y instead on $\hat{X}$—the predicted value of X from a regression of X on G – *will* yield a consistent estimate for β, because $\hat{X}$ is independent of U.^3^^,^^4^

TSLS relies on individual-level data, but the sharing of such data is often impractical. In recent years, it has become much more common to attempt MR analyses using summary data estimates of SNP–exposure and SNP–outcome associations gleaned from two independent but homogeneous study populations. The SNPs in question are usually identified as genome-wide significant ‘hits’ in distinct genomic regions via a genome-wide association study (GWAS) for the exposure. This is referred to as ‘two-sample summary data MR’.

Let $\pi_{j}$ and $\Gamma_{j}$ represent the true association for SNP $G_{j}$ in G with the exposure and the outcome, respectively. From Equations (1) and (2), we can link the j’th SNP-outcome association to the j’th SNP–exposure association via the model

(3) Γ^j = βπ^j. 

It follows that the Wald estimator ${\hat{\beta }}_{j}\;=\;\frac{{\hat{\Gamma }}_{j}}{{\hat{\pi }}_{j}}$ is a consistent estimate for β^5^^,^^6^ where ${\hat{\Gamma }}_{j}$ and ${\hat{\pi }}_{j}$ are estimates from OLS estimation of

$$Y=\Gamma_{0}+\Gamma_{j}G_{j}+\epsilon_{y,j}$$

and

$$X=\pi_{0}+\pi_{j}G_{j}+\epsilon_{X,j}$$

When the SNPs are uncorrelated, taking an inverse variance weighted (IVW) average of the ratio estimates will yield an overall estimate for β, ${\hat{\beta }}_{IVW}$, that closely approximates the TSLS estimate that would have been obtained if individual-level data were available.^7^

### Detecting ‘weak’ instruments and ‘invalid’ instruments in MR

If assumptions IV1–IV3 are fulfilled for all SNPs in G, and linear Equations (1) and (2) hold, then either a TSLS or IVW analysis (with uncorrelated SNPs) will consistently estimate the causal effect.^8–10^ In order to satisfy IV1, the SNPs in G should strongly predict the exposure X. This can be quantified using the F-statistic from the first stage regression of X on G. Using instruments that are jointly only weakly associated with the exposure (i.e. which have a small F-statistic) will result in weak-instrument bias.^11^

Second, SNPs should not exert a direct effect on Y, i.e. they should not affect Y other than through X. Any such effect would represent a violation of IV2. Horizontal pleiotropy, where the genetic variants used as instruments have an effect on the outcome that is not through the exposure of interest, is a violation of the exclusion restriction and could easily be responsible for such a violation in the MR setting.^10^^,^^12–14^ The SNPs should also not be confounded by any variables that also influence the outcome. Any confounding of this nature would be a violation of assumption IV3. A violation of either assumptions IV2 or IV3 is likely to lead to bias and potentially erroneous conclusions in both the TSLS and IVW estimates.^4^ The presence of potential pleiotropic effects can be evaluated using the Sargan test^15^^,^^16^ using individual-level data and Cochran’s Q statistic^17–19^ using summary data. The causes and consequences of pleiotropy in MR are described in detail elsewhere.^1^^,^^9^^,^^10^^,^^13^^,^^14^^,^^20^

In addition to assumptions IV1–IV3, there are additional assumptions and considerations that apply to all instrumental variable estimation, including MR and MVMR. These included the assumptions of linearity and homogeneity that are in many settings required for obtaining a point estimate of the causal effect. However, if this assumption is violated, the causal null will still be respected and it will still be possible to identify whether the exposures are causally associated with the outcome.^21–23^ Throughout this analysis, we assume linearity of the relationship between the exposures and the outcome although, if this assumption did not hold, the same issues would apply to MVMR as apply in MR analysis, which are discussed in detail elsewhere.^24^^,^^25^

## MVMR

**Figure 2.**
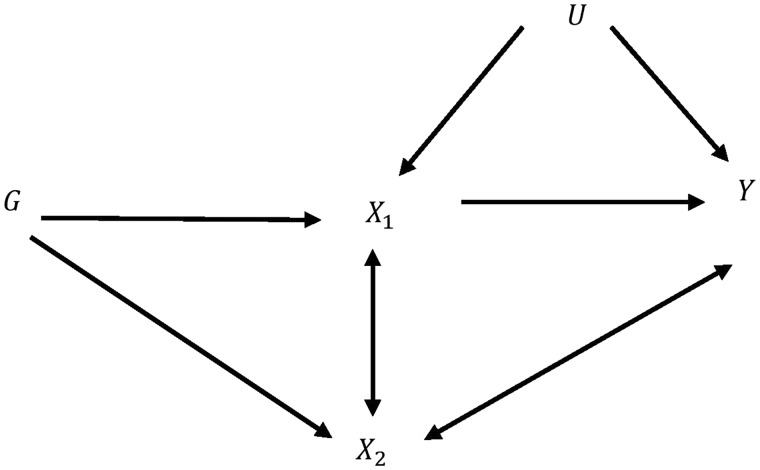
Hypothesized relationship between genetic variant(s) *G*, modifiable exposures, *X*_1_, *X*_2_ and outcome *Y* in the presence of unobserved confounder *U*. Bi-directional arrows represent possible violations of the IV assumptions induced by *X*_2_ that are explored in this paper.

MR can be extended to estimate the effect of multiple exposure variables on an outcome^1^ and is particularly useful in cases where a standard MR analysis would fail due to violation of assumptions IV2 and IV3. It is also useful in cases where two or more correlated exposures are of interest and may help to understand whether both exposures exert a causal effect on the outcome, or whether one in fact mediates the effect of the other on the outcome.^26^^,^^27^ MVMR requires a set of SNPs, G, that are associated with the exposure variables but do not affect the outcome other than through these variables. In the same way as standard (single-variable) MR, these SNPs can be used to predict each of the exposure variables in the model and these predicted values can be used to estimate the effect of the exposures on the outcome in a multivariable regression analysis. The setup for MVMR is illustrated for an analysis involving two exposure variables ($X_{1}$ and $X_{2}$) in Figure 2. The arrows linking $X_{1}$ with $X_{2}$ and $X_{2}$ with Y have been left bi-directional to acknowledge the fact that many underlying causal relationships are possible, i.e. they could point in either direction or be completely absent. Indeed, many of these options will be subsequently explored.

Although it is the simplest possible MVMR setting, two exposures suffice to illustrate all the scenarios and ideas described in this paper. From Figure 2, we can write the following general model linking Y, $X_{1}$, $X_{2}$ and U:

(4) Y = β0 + β1X1 + β2X2 + U + vy. 

For example, suppose that $X_{1}$ and $X_{2}$ are in fact independent given *G* (so there is *no* arrow in Figure 2 between $X_{1}$ and $X_{2}$) and $X_{2}$ affects Y independently of $X_{1}$ (so that there is a *direct arrow* from $X_{2}$ to Y). If true, then Equations (5) and (6) for $X_{1}$ and $X_{2}$ would, jointly with Equation (4), describe the data:

(5) X1 = π01 + π1G + U + vx1

(6) X2 = π02 + π2G + U + vx2. 

The purpose of an MVMR analysis is to determine the direct causal effect of both $X_{1}$ and $X_{2}$ on the outcome Y, when conditioned on one another. Without loss of generality, we will focus primarily on the effect of $X_{1\;}$(and the parameter $\beta_{1})$ with the direct effect of $X_{2}$ on Y denoted by $\beta_{2}\;$being of secondary importance.

With individual-level data, TSLS can be implemented with multiple exposure variables, regressing each exposure on the full set of SNPs to yield genetically predicted estimates for $X_{1}$ and $X_{2}$. The outcome *Y* can then be regressed on these predicted estimates for $X_{1}$ and $X_{2}$ jointly to obtain consistent estimates of $\beta_{1}$ and $\beta_{2}$. This can be conducted by simply using the *ivreg2* command in Stata or *ivpack* in R.

In the two-sample summary data setting, Burgess *et al.*^1^^,^^2^ show how MVMR can be implemented using summary data estimates of the association between SNP j (out of L) and the outcome, ${\;\hat{\Gamma }}_{j}$; exposure $X_{1}$, ${\hat{\pi }}_{1j}$; and exposure $X_{2}$, ${\hat{\pi }}_{2j}$, by fitting the following model:

(7)  Γ^j = β1π^1,j + β2π^2,j + ϵj.  

This is a straightforward generalization of the IVW estimation framework.

### Important considerations

To conduct an MVMR analysis, it is necessary to have at least as many genetic instruments as there are exposures to be instrumented in the model; this is true regardless of whether single-sample or two-sample summary data are used. It is possible to include genetic instruments that are associated with more than one exposure variable, providing all of those exposure variables are included in the estimation. Instruments must not, however, exert a direct effect on the outcome, except through the included exposures. There is no benefit to excluding instruments that are only strongly associated with one exposure, as this will lead to a loss of precision in the estimates obtained. This also avoids any potential bias that could arise due to selecting instruments based on their strength.^11^

## What quantities do MR and MVMR estimate?

MR and MVMR target different causal effects of the exposure on the outcome. In general, MR estimates the *total* effect of the exposure on the outcome, whereas MVMR estimates the *direct* effect of each exposure on the outcome.

**Figure 3.**
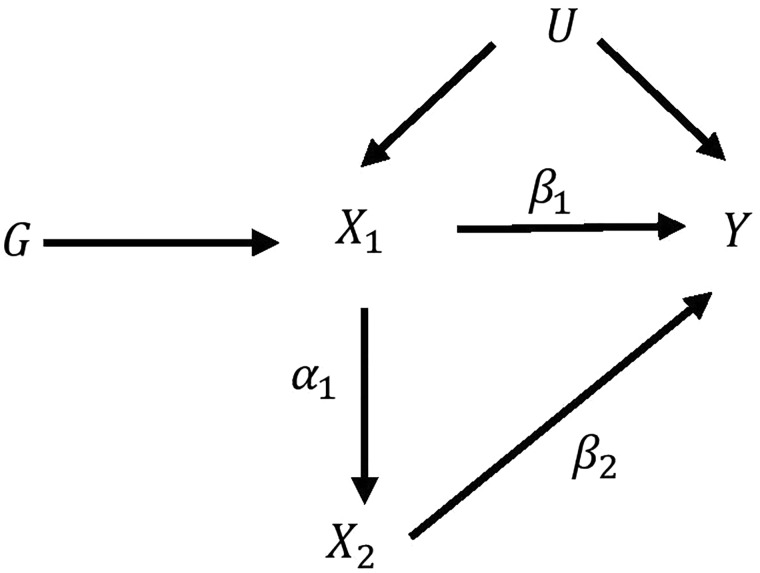
Illustration of the direct effect and total effect of *X*_1_ on the outcome *Y*.

For example, if Figure 3 describes the truth, the total effect of exposure $X_{1}$ on the outcome is the effect of $X_{1}$ on the outcome Y directly plus the effect of $X_{1}$ on Y via$\;X_{2}$, and is equal to $\beta_{1}\;+\;\alpha \beta_{2}$. The direct effect of the exposure $X_{1}$on the outcome Y is the effect $X_{1}$ has on Y not via any other exposure variables included, and so is equal to $\beta_{1}.$ Whether or not these effects differ in general depends on the underlying relationship between the exposures and between each exposure and the outcome. If there is no effect of $X_{1}$ on $X_{2}$ or of $X_{2}$ on Y, i.e. either α or $\beta_{2}$ is equal to zero, these effects will be the same.

To highlight the potential differences between MR and MVMR, and the potential benefits of MVMR, we now consider the application of MVMR to four different scenarios that are commonly encountered, or at least suspected, in epidemiological studies.

Each of these scenarios represents a situation in which conventional univariable MR would produce consistent results, given the correct set of SNPs, but in which MVMR may estimate a different causal effect and provide benefits when in fact some of the SNPs may have effects on more than one exposure (thus making them invalid instruments for a univariable MR analysis). In the first scenario, $X_{2}$ is a *confounder* of the relationship between $X_{1}$ and Y, i.e. there is a direct causal path from $X_{2}$ to $X_{1}$ and from $X_{2}$ to Y. Along with Equation (4), Equation (6) above and Equation (8) below underlie the individual-level data:

(8) X1 = π1G + α2X2 + U + vx1.  

In the second scenario, $X_{2}$ is a *collider* of the relationship between $X_{1}$ and Y, i.e. there is a direct causal path from $X_{1}$ to $X_{2}$ and from Y to $X_{2}$. When an exposure and outcome both influence another variable, controlling for that variable in conventional analysis will introduce bias into the observed association between the exposure and the outcome.^28^ This form of bias can also be understood as a form of selection bias that would occur if inclusion in the sample was dependent on the value of $X_{2}$.^29^ Along with Equation (4) (with $\beta_{2}$ set to 0), Equation (5) above and Equation (9) below are used to generate the individual-level data:

(9) X2 = π2G + α1X1 + γyY + U + vx2. 

In the third scenario, $X_{2}$ is an independent *pleiotropic* pathway from G to Y. This corresponds to the scenario first described in the previous section. Along with Equation (4), Equations (5) and (6) above are used to generate the individual-level data.

In the fourth scenario, $X_{2}$ is a *mediator* of the relationship between $X_{1}$ and Y. Along with Equation (4), Equation (5) above and Equation (10) below are used to generate the individual-level data:

(10) X2 = π2G + α1X1 + U + vx2.  

**Figure 4.**
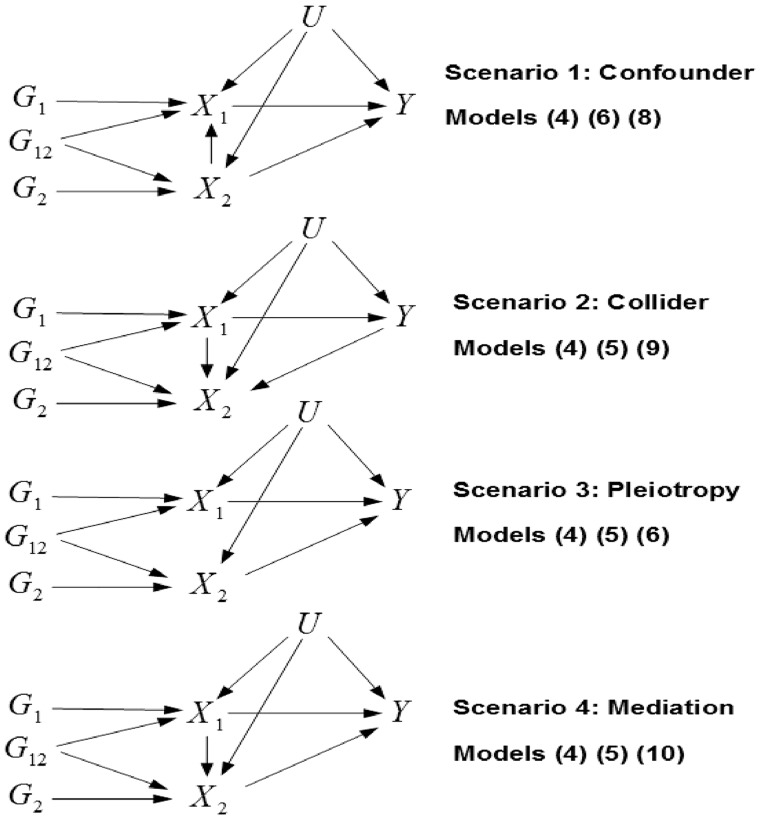
Causal diagrams for scenarios 1–4. Models referred to are the equations above that would give the same relationship between the instruments, exposures and outcome. *G*_1_, *G*_2_ and *G*_12_ are subsets of the full set of SNPs *G* that affect *X*_1_, *X*_2_ and both exposures, respectively.

Each of these scenarios is shown in Figure 4.

### Simulations

Datasets of 10 000 individuals are simulated under all four scenarios discussed using L = 30 genetic variants. The variants are assumed to be uncorrelated but, for added realism and complexity, are further subdivided into three categories:

- 10 SNPs that only predict $X_{1}$: $G_{1}$ (with a non-zero $\pi_{1}$ element but zero $\pi_{2}$ element);
- 10 SNPs that only predict $X_{2}$: $G_{2}$ (with a non-zero $\pi_{2}$ element but zero $\pi_{1}$ element);
- 10 SNPs that predict $X_{1}$ and $X_{2}$: $G_{12}$ (with non-zero $\pi_{1}$ and $\pi_{2}$ elements).

G therefore represents the complete vector $\left(G_{1},G_{2},G_{12}\right)$. For each scenario, the causal parameter of interest, $\beta_{1}$, is set to 1.

For each scenario, we estimate the causal effect$s\;\beta_{1}$ and $\beta_{2}\;$of $X_{1}$ and $X_{2}$ on Y, using a range of estimation methods. With *single-sample individual-level data*, we implemented:

- OLS, both for $X_{1}\;$and $X_{2}$ individually (i.e. univariable regressions) and together (i.e. a multivariable regression);
- MR for $X_{1}$ and $X_{2}$ individually, each time using all the available SNPs as instruments;
- MVMR including both $X_{1}$ and $X_{2}$ in the same analysis;
- MR for$\;X_{1}$ and $X_{2}$ individually using only the SNPs that are valid instruments for that exposure ($G_{1}$ and $G_{2},$ respectively).

With *two-sample summary-level data*, we implemented:

- MR for $X_{1}$ and $X_{2}$ individually using all of the instruments available;
- MVMR including both $X_{1}$ and $X_{2}$;
- MR for $X_{1}$ and $X_{2}$ individually using only the SNPs that are valid instruments for the exposure.

All estimation methods are described in Supplementary Table 1, available as Supplementary data at *IJE* online. In all of the scenarios considered, the exposure variables are strongly predicted by the instruments and the instruments have no additional pleiotropic effects on the outcome, other than through the exposures included in the model.

### Results

Focusing our attention on exposure $X_{1}$, the results from these simulations show that MVMR always gives an unbiased estimate of the *direct* effect of $X_{1}$ on the outcome. In the hypothetical case in which only the valid SNPs for $X_{1\;}$ ($G_{1}$) are used as instruments in a single-variable MR, the estimated effect of $X_{1}$ on Y is the *total* effect of a change in $X_{1}\;$on the outcome. Whether the direct or total effect is of more interest to practitioners will depend on the particular situation being considered. In many of the scenarios explored, the direct effect equals the total causal effect; however, when $X_{2}$ is a mediator of the relationship between $X_{1}$ and the outcome, the direct and total effects of $X_{1}$ may be substantially different. In this scenario, MVMR is not a form of mediation analysis, but instead estimates the direct effect of the exposure on the outcome that does not act via the mediator. The results from the simulations are given in Supplementary Table 2, available as Supplementary data at *IJE* online, and a summary table of what is estimated by each method in each scenario is given in Table 1.

**Table 1.** Summary of estimated effects for $\beta_{1}$

	Scenario/which estimand is targeted?			
Method	I	2	3	4
Individual-level data				
OLS	x	x	x	x
Univariate MR	x	Direct/total	x	x
MVMR	Direct/total	Direct/total	Direct/total	Direct
Univariate MR—subset of SNPS	Direct/total	Direct/total	Direct/total	Total
Two-sample summary data analysis				
Univariate MR	x	Direct/total	x	x
MVMR	Direct/total	Direct/total	Direct/total	Direct
Univariate MR—subset of SNPS	Direct/total	Direct/total	Direct/total	Total

When each method of estimation estimates the direct and total effects for $\beta_{1}$ in each of the scenarios considered.

An ‘x’ represents a biased method of estimation.

When conducting the univariable MR estimation with a subset of the SNPs in G, we have, for illustration, assumed ‘oracle’ knowledge on which SNPs are valid instruments for each exposure. This will, of course, not be possible in practice For example, in Scenario 1, if we select SNPs because they are associated with a $X_{1}$, we will select the entire set *G*, but this will include the subset $(G_{2},G_{12}$), which exerts pleiotropic effects on the outcome *Y* and thus biases the analysis. Table 1 indeed shows that, when all SNPs in *G* are used for a univariable MR analysis, it will deliver a biased and inconsistent estimate of the total causal effect of $X_{1}$ on *Y* in Scenarios 1, 3 and 4. MVMR, by contrast, will then provide a consistent estimator of the direct effect of the exposure on the outcome; the consistency of IV analysis under a range of scenarios that include those discussed here has been proved elsewhere.^3^^,^^4^^,^^30^ These simulation results also highlight that MVMR does not introduce bias into the results when $X_{2}$ is a collider of the relationship between $X_{1}$ and Y. This is because the predicted value of$\;X_{2}$,$\;{\hat{X}}_{2}$, which is not dependent on the outcome, is used in the analysis. Of course, adjusting directly for $X_{2}$, rather than ${\hat{X}}_{2}$, would bias the analysis. This is an important benefit of MVMR.

## Testing the assumptions of MVMR

In the simulations above, we assumed, for clarity, that the instruments were both strong and valid for the purposes of an MVMR analysis. However, violation of these assumptions can give misleading results in practice, so it is necessary to test these assumptions. We now describe how instrument strength and validity can be scrutinized for an MVMR analysis in the individual and two-sample summary data settings.

In addition to assumptions IV1–IV3, there are additional assumptions and considerations that apply to all instrumental variable estimation, including MR and MVMR. These included the assumptions of linearity and homogeneity, which is in many settings required for obtaining a point estimate of the causal effect. Increasing the number of exposures in two-sample MVMR will make this a stronger assumption due to the increased number of SNPs and exposures. When implementing MVMR analysis, this limitation should be considered and weighed against the benefits when deciding how many exposures to include in the analysis. Another additional assumption, particularly relevant to two-sample MVMR analysis, is that all data are drawn from the same underlying population. Throughout our analysis, we assume this to hold. The requirement for and issues surrounding this assumption are detailed elsewhere.^31^^,^^32^

### The individual-level data MVMR setting

#### Instrument strength

**Figure 5.**
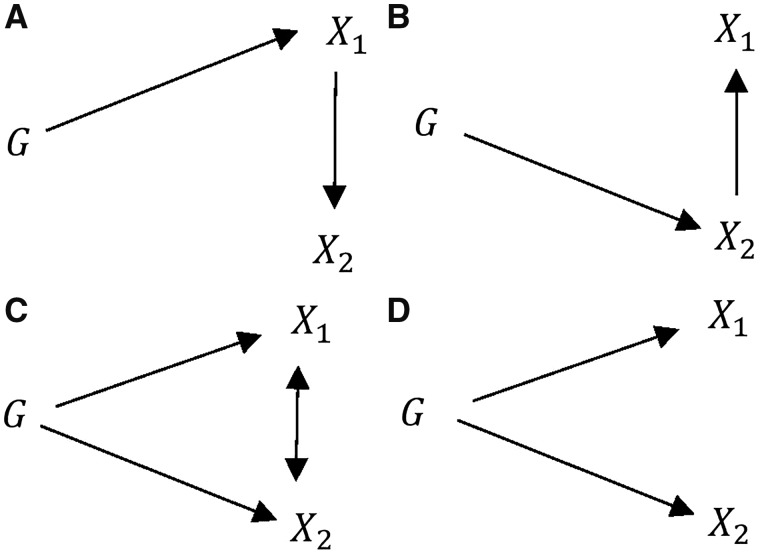
Potential setups of instruments and exposures. In (**A**) and (**B**), the exposures are individually strongly predicted but are not jointly predicted. In (**C**), the exposures are individually strongly predicted but weakly predicted in a joint sense. In (**D**), the exposures are individually and jointly strongly predicted. Specifically: (**A**) *G* predicts *X*_1_, which is a predictor of *X*_2_. (**B**) *G* predicts *X*_2_, which is a predictor of *X*_1_. (**C**) *G* predicts *X*_1_ and *X*_2_, which are highly correlated. (**D**) *G* predicts *X*_1_ and *X*_2_, which are uncorrelated (given *G*).

In any MR analysis, the set of genetic instruments *G* must be strong in order to avoid weak-instrument bias (assumption IV1). In single-variable MR analysis, weak instruments will bias the estimated results in the direction of the observational estimate; however, in MVMR analysis, it is not clear what direction the bias of the estimation result for each exposure will take as a result of weak instruments.^33^ It is therefore important to test the strength of the instruments in any MVMR analysis; however, the assessment of instrument strength is more complicated. It is necessary for *G* to strongly predict both $X_{1}$ and $X_{2}$ (as quantified by strong F-statistics), but not sufficient. In addition, *G* must also *jointly* predict both $X_{1}$ and${\;X}_{2}$, i.e. once the secondary exposure $X_{2}\;$has been predicted using G, G must *still* be able to predict the primary exposure $X_{1}$. Figure 5 illustrates three scenarios (A–C) in which this may not be the case, even when both exposures appear to be strongly predicted individually by G and a fourth scenario (D) in which both exposures are strongly predicted.

Joint strength can be assessed using the Sanderson–Windmeijer conditional F-statistic,^33^${\;F}_{c}$, that is available as part of ivreg2 in Stata. $F_{c}$ is calculated in the following manner:

- $X_{2}$ is regressed on the full set of genetic instruments (and any control variables included in the estimation) and the predicted value of $X_{2}$, ${\hat{X}}_{2}$, is calculated;
- $X_{1}$ is then regressed on ${\hat{X}}_{2}$ (and any control variables) to yield the TSLS estimate $\hat{\delta }$ and the residual error terms $X_{1}-\;\hat{\delta }X_{2}$ are saved;
- the errors are then regressed on the full set of instruments (and any control variables); the conditional F-statistic is obtained as the F-statistic for the effect of the instruments in this regression;
- the conditional F-statistic must be adjusted for a degrees-of-freedom correction, and can be compared with the conventional weak-instrument critical values.^34^

For multiple exposure variables, the first step is repeated for each of the exposures and all of these predicted values are included in the regression in the second step. This F-statistic can be compared with the standard critical values for weak instruments; therefore, if the conditional F-statistic for all of the exposure variables is larger than the rule-of-thumb value of 10, then the instruments can be considered adequately strong for the purposes of MVMR.

#### Instrument validity

If no pleiotropy exists amongst the genetic variants, then each one should identify the same causal parameter. This can be evaluated using the Sargan test.^15^ Specifically, it tests whether the instruments can explain any of the variation in the outcomes that has not been explained by the value of the exposure variables. It is calculated by the following steps:

- regress the outcome *Y* on the exposures using TSLS to yield causal estimates ${\hat{\beta }}_{1}$ and ${\hat{\beta }}_{2};$
- calculate the residual error term $Y-({\hat{\beta }}_{1}X_{1}+{\hat{\beta }}_{2}X_{2})$ and then regress the residuals on the full set of instruments; the Sargan test is then the sample size times the R^2^ of this regression;
- evaluating with the Sargan statistic with respect to a $\chi^{2}$ distribution with degrees of freedom equal to the number of instruments minus the number of predicted exposure variables (i.e. the null hypothesis that all of the instruments are valid).^4^

This test is available as part of the ivreg2 command in Stata and the ivpack package in R. In order to conduct this test, the model must be over-identified, i.e. there must be more instruments than exposure variables (so that the degrees of freedom of the $\chi^{2}\;$test is positive).^35^ This ‘global’ test does not give any indication as to which of the genetic instruments are invalid if the test rejects the null. However, alternative methods of estimation can be used to estimate the causal effects as long as at least 50% of the SNPs do not have a pleiotropic effect on the outcome.^36^^,^^37^

### The two-sample summary data setting

Assessment of instrument validity and strength is apparently yet to be described in the two-sample summary data setting that is relevant to the majority of contemporary MR studies, and consequently it is not implemented in any standard software. We therefore describe the necessary procedures in fine detail so that they can be confidently implemented by others.

#### Assessing instrument strength: heterogeneity is ‘good’

Suppose that all of the genetic instruments predict both exposure variables, so that Equations (4), (5) and (6) hold, but there are at least two elements of $\pi_{1}$and $\pi_{2}$ in Equations (5) and (6) that differ. If true, then the model will be *at least exactly identified*, i.e. there will be at least as many independent genetic instruments (i.e. two) as there are exposure variables to be instrumented. This implies that

(11) X1 = δ0 + δX2 + u1.X2=π0+π2G+u2  

must be over-identified (or equivalently miss-specified), because $X_{2}$ cannot then be simply a scalar multiple, δ, of $X_{1}$. Therefore, we can test for under-identification in our estimation model by testing for over-identification in Equation (11) using the Sargan test as described above. The equivalence of this test with the Sanderson–Windmeijer approach has been shown formally elsewhere.^38^ The null of this Sargan test is that of under-identification.

Extending this to two-sample analysis, ${\;\hat{\pi }}_{1,j}={\;\delta \hat{\pi }}_{2,j}+\varepsilon_{1,j}$ is analogous to Equation (11) estimated by IV using individual-level data with ${\;\hat{X}}_{2}$ predicted using *G*; therefore, it should be possible to test for under-identification in two-sample MVMR estimation by testing for over-identification in the model ${\;\hat{\pi }}_{1,j}={\delta \hat{\pi }}_{2,j}+v$. We recommend that this test is conducted using a modified version of Cochran’s Q statistic, as shown in Equation (12) below:

(12) $$Q_{x_{1}}=\sum_{j=1}^{L}\left(\frac{1}{\sigma_{x1j}^{2}}\right){\left({\hat{\pi }}_{1j}-{\hat{\delta }\hat{\pi }}_{2j}\right)}^{2}.$$

The variance term for $Q_{x_{1}}$, $\sigma_{x1j}^{2}=\sigma_{1j}^{2}+{\hat{\delta }}^{2}\sigma_{2j}^{2}-2\hat{\delta }\sigma_{12j}$, where $\sigma_{1j}^{2}$ is the variance of ${\hat{\pi }}_{1,j}$, $\sigma_{2j}^{2}\;$is the variance of ${\hat{\pi }}_{2,j}$, $\sigma_{12j}$ is the covariance of ${\hat{\pi }}_{1,j}$ and ${\hat{\pi }}_{2,j}$, and $\hat{\delta }$ is an efficient estimator for δ. Estimation of the $\sigma_{x1j}^{2}$ terms in practice depends on the type of model used to obtain ${\hat{\pi }}_{1,j}$ and${\hat{\;\pi }}_{2,j}$. When each exposure is regressed on the entire set of SNPs simultaneously (i.e. via multivariable regressions with an intercept):

$$\sigma_{1j}^{2}\;=\;\frac{{\left(G^{T}G\right)}_{jj}^{-1}}{n}\sum_{i=1}^{n}{\hat{v}}_{1i}^{2},\;\;\sigma_{2j}^{2}\;=\;\frac{{\left(G^{T}G\right)}_{jj}^{-1}}{n}\sum_{i=1}^{n}{\hat{v}}_{2i}^{2}\;\;\;and\;\sigma_{12j}\;=\;\frac{{\left(G^{T}G\right)}_{jj}^{-1}}{n}\sum_{i=1}^{n}{\hat{v}}_{1i}\;{\hat{v}}_{2i},$$

where n is the number of subjects and $\left({\hat{v}}_{1i},{\hat{v}}_{2i}\right)$ are the estimated residuals from these regressions. If ${\hat{\pi }}_{1j}$ and ${\hat{\pi }}_{2j}$ are obtained separately (i.e. via univariable regressions with an intercept), then the error terms are obtained from the equivalent expressions are

$$\sigma_{1j}^{2}\;=\;\frac{{\left(G_{j}^{T}G_{j}\right)}^{-1}}{n}\sum_{i=1}^{n}{\hat{v}}_{1ij}^{2},\;\sigma_{2j}^{2}\;=\;\frac{{\left(G_{j}^{T}G_{j}\right)}^{-1}}{n}\sum_{i=1}^{n}{\hat{v}}_{2ij}^{2}\;\;\;and\;\sigma_{12j}\;=\;\frac{{\left(G_{j}^{T}G_{j}\right)}^{-1}}{n}\sum_{i=1}^{n}{\hat{v}}_{1ij}\;{\hat{v}}_{2ij}.\;$$

Respectively, ${\hat{v}}_{1ij}$ and ${\hat{v}}_{2ij}$ are the estimated residuals from the *j*’th regression.

Under the null hypothesis that the instruments do not contain enough information to predict both exposure variables, $Q_{x_{1}}$ will be asymptotically $\chi_{L-1}^{2}$ distributed when δ is estimated using an asymptotically efficient estimator, where L is the number of instruments. Rejection of the null hypothesis (i.e. detection of ‘heterogeneity’) indicates that the model we wish to estimate *is* identified for $X_{1}$.

All the above can be repeated for $X_{2}$ by swapping the roles of ${\;\hat{\pi }}_{1}$and ${\;\hat{\pi }}_{2}\;$and calculating an equivalent Q statistic for $X_{2}$, $Q_{x_{2}}\;$say. If both $Q_{x_{1}}\;$and $Q_{x_{2}}\;$are larger than the chosen critical value, then the null hypothesis of under-identification can be rejected and the test suggests that the instruments can predict variation in both exposures. Table 2 shows the distribution of $Q_{x_{1}}\;$and $Q_{x_{2}}$for four different scenarios with two exposure variables and L = 100 SNPs. $X_{1}$ and $X_{2}$ are both functions of a set of SNPs and independent confounding variables. In the first simulation, the model has been set up as given in Scenario 3 in Figure 4 and in Figure 5D with each of the exposure variables predicted by a set of SNPs and a common confounding variable. This model is identified as both exposure variables can be predicted by the set of instruments. In the second and third simulations, the model has been set up in the same way but with no effect of the SNPs on either $X_{1}$ or $X_{2}$, respectively, i.e. the model is under-identified with one of the exposure variables not being predicted by the instruments in each case. In the final simulation, the model has been set up with the effect of the SNPs on the exposures as given in Figure 5A and a common confounder. This setup leads to neither exposure being predicted by the SNPs when they are both included in an MVMR estimation, as the SNPs in the model cannot predict both of the exposure variables jointly. The results from these simulations show that this test has the required distribution under the null hypothesis.

**Table 2.** The distribution of the modified Q statistic as a test for under-identification

	$Q_{x_{1}}$			$Q_{x_{2}}$		
	Mean	Std. dev	Rej. rate (%)	Mean	Std. dev	Rej. rate (%)
$x_{1}$ strongly identified	1 953 018	26 441	100	1 952 738	247 541	100
$x_{2}$ strongly identified						
$x_{1}$ unidentified	99.3	14.6	6.2	379 282	135 506	100
$x_{2}$ strongly identified						
$x_{1}$ strongly identified	453 058	1 607 136	100	100.2	14.6	6.6
$x_{2}$ unidentified						
$x_{1}$ strongly identified	100.2	14.6	6.6	100.2	14.6	6.6
$x_{2}$ strongly identified						
Jointly unidentified $x_{1}=\delta x_{2}$, δ=1						

*N* = 50 000. Repetitions = 1000, 100 SNPs as instruments. Rejection rates give the proportion of times each Q statistic is larger than the 95th percentile of a Chi-squared distribution on 99 degrees of freedom (123.2).

#### Testing instrument validity: heterogeneity is ‘bad’

Cochran’s Q statistic for the regression of interest has been proposed as a method for identifying the presence of invalid instruments (e.g. due to horizontal pleiotropy) in two-sample summary data MR analysis, with a single exposure.^19^ Specifically, if all instruments are valid IVs, and the modelling assumptions necessary for two-sample MR are satisfied, then each genetic instrument should give the same estimate of the effect of the exposure on the outcome. Excessive heterogeneity in the causal-effect estimates obtained by each SNP individually now becomes an indicator of invalid instruments. We propose testing for invalidity in two-sample summary data MVMR using an adjusted version of the Cochran Q statistic given by:

(13) $$Q_{A}=\sum_{j=1}^{L}\left(\frac{1}{\sigma_{Aj}^{2}}\right){\left({\hat{\Gamma }}_{j}-\left({\hat{\beta }}_{1}{\hat{\pi }}_{1j}+\;{\hat{\beta }}_{2}{\hat{\pi }}_{2j}\right)\right)}^{2}.$$

Where $\sigma_{Aj}^{2}=\sigma_{yj}^{2}+{\hat{\beta }}_{1}^{2}\sigma_{1j}^{2}+{\hat{\beta }}_{2}^{2}\sigma_{2j}^{2}+2{\hat{\beta }}_{1}{\hat{\beta }}_{2}\sigma_{12j}$. To clarify, $\sigma_{yj}^{2}$ is the variance of${\;\hat{\Gamma }}_{j}$, and ${\hat{\beta }}_{1}$ and ${\hat{\beta }}_{2}$ are efficient estimates of $\beta_{1}$ and $\beta_{2}$ [e.g. as obtained from fitting Equation (7)]. Under the null hypothesis that the genetic instruments do not have pleiotropic effects on the outcome, $Q_{A}\;$is asymptotically $\chi^{2}$ distributed with (L - 2) degrees of freedom. The standard implementation of Cochran’s Q would merely have a weighting of${\;\sigma }_{yj}^{2}$, and is not therefore asymptotically $\chi^{2}$ distributed. It is a straightforward generalization of the adjusted Q statistic recently proposed by Bowden *et al*. in the univariable MR setting.^18^ Excessive heterogeneity in $Q_{A}$ therefore brings assumptions IV2 and IV3 into doubt.

**Figure 6.**
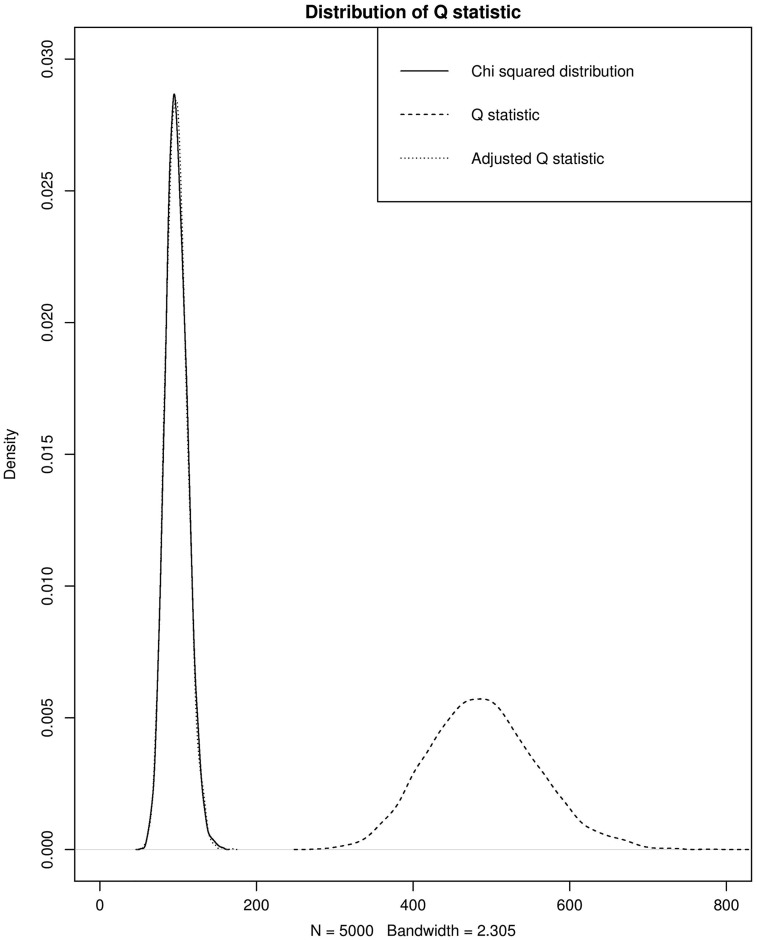
The distribution of the adjusted and standard Q statistics under the null hypothesis of no heterogeneity. 5000 repetitions, 100 SNPs. Here, β_1_ = β_2_ = 1. σ_1j_ 2 = σ_2j2_ = 0.02, σ_12j_ = 0 for a.

Figure 6 shows the distribution of $Q_{A}$ compared with the standard Q statistic and a $\chi^{2}$ distribution with 98 degrees of freedom for a model with two exposure variables and 100 genetic instruments. For simplicity, the estimated effects of the SNPs on the exposures each have a common variance of 0.02 and have a common covariance of 0. $Q_{A}$ is seen to have the correct distribution under the null hypothesis of no pleiotropy in the model.

We suggest updating the two-sample causal estimates in an iterative process using weights derived from the initial estimates of the causal effects, which is referred to as ‘modified iterative’ weighting in Bowden *et al.*^18^ within the context of a univariate MR analysis. Further work is required to fully investigate the effect of this and to understand how the fully analytical solution discussed in,^18^ which finds that the causal estimate that directly minimizes an equivalent Q statistic could be extended to the multivariable case but, if done so, this could help to mitigate the effect of weak-instrument bias.

### Approximating $Q_{x1,\;}Q_{x2}$ and $Q_{A}$ with incomplete information

The covariance vector $\sigma_{12j}$ that is necessary for correct specification of $Q_{x1,\;\;}Q_{x2}$ and $Q_{A}$ can only be calculated from the individual participant data. If this information is not available, one solution would be to ensure that $\sigma_{12j}$ is zero, by estimating the genetic associations with each exposure *and* the outcome in separate samples. This would correspond to a ‘three-sample’ summary data MR analysis when two exposures constitute the MVMR analysis.

Another pragmatic solution would be to assume that each $\sigma_{12j}$ term is zero. This will give a good approximation for $Q_{x1\;}$and $Q_{x2}$ whenever $\delta \sigma_{12j}$ is small and for $Q_{A}\;$whenever ${\hat{\beta }}_{1}{\hat{\beta }}_{2}\sigma_{12j}$ is small.

## Application to education, cognitive ability and body mass index (BMI)

In this section, we apply the methods discussed above to investigate whether there is evidence for a causal effect of education and cognitive ability on BMI using data from UK Biobank. Education and cognitive ability have both been found to be associated with BMI, with higher levels of education and cognitive ability being associated with lower levels of BMI.^39–42^ However, there is also a high level of correlation observed between completed education and measured cognitive ability; therefore, it is not clear whether, once this correlation has been controlled for, both education and cognitive ability have a causal effect on BMI.^39^

### Data

UK Biobank recruited 502641 individuals aged 37–73 years between 2006 and 2010 from across the UK. Individuals were invited to a clinic where they answered a questionnaire and interview about a range of health topics and provided anthropomorphic measurements and gave samples of blood, urine and saliva. This study has been described in full previously.^43^

Individuals in UK Biobank were asked to report the highest educational qualification they had obtained. For each individual, we assigned an age at which they left education based their reported qualification. A breakdown of educational qualifications and associated ages across the cohort is given in Supplementary Table 3, available as Supplementary data at *IJE* online.

Cognitive ability was measured among a subset of the UK Biobank participants as the number of correct answers recorded in a series of 13 questions designed to measure cognitive ability that where completed as part of the initial clinic. The cognitive ability variable was then standardized to have mean zero and variance 1. BMI was calculated based on the height and weight of the individuals in the sample. Throughout the analysis, we analysed this variable on the natural log scale because of its skewed distribution.

### Analysis

We first conducted MR analyses for the effect of education and cognitive ability on BMI separately using single-variable MR. A single composite instrument for education was created using the polygenic score of 74 SNPs from a recent GWAS of educational attainment.^44^ A single composite instrument for cognitive ability was created using the polygenic score of 18 SNPs from a recent GWAS of cognition.^45^ As this GWAS was conducted using the interim release of UK Biobank, we restricted our analysis to individuals not included in the interim release.

We then conducted a multivariable MR analysis of the effect of education and cognitive ability on BMI. This analysis included both the composite instruments for education and cognitive ability used in the single-variable MR analyses.

The results from this analyses, along with a multivariable OLS regression of BMI on education and cognitive ability, are given in Table 3. The OLS results show that each extra year of education is associated with a decrease in BMI. MR and MVMR results suggest a causal effect in the same direction, but with a larger magnitude. The results for cognitive ability are more mixed, with no association seen in the OLS results, a negative total effect of cognitive ability on BMI in the MR analysis and potentially a positive direct effect of cognitive ability on BMI observed in the MVMR analysis. Our empirical and theoretical investigation helps to clarify why the the high level of correlation between education and cognitive ability would lead to the conclusion that there is a negative effect of cognitive ability on BMI in MR analysis. The MVMR results show that, if anything, the direct effect of increasing cogntive ability is to increase BMI. These results highlight the potential benefits of MVMR. However, before giving much credence to this result, it is necessary to assess the strength of our SNPs to jointly predict education and cognitive ability.

**Table 3.** The effect of education and cognitive ability on BMI

		OLS		MR	
		Single variable	Multivariable	Single variable	Multivariable
Age completed education	Effect	–0.008	–0.008	–0.028	–0.044
	Std. error	(0.0003)	(0.0003)	0.005	0.013
	95% CI	[–0.0095, –0.0074]	[–0.0085, –0.0074]	[–0.0391, –0.0179]	[–0.0704, –0.0187]
	F-statistic			188.2	195.0
	S-W F-statistic				35.7
Standardized cognitive ability score	Effect	–0.006	0.0001		0.048
	Std. error	(0.0007)	(0.0007)	0.008	0.025
	95% CI	[–0.0078, –0.0051]	[–0.0013, 0.0014]	[–0.0380, –0.0082]	[–0.001, –0.098]
	F-statistic			542.2	309.7
	S-W F-statistic				37.0

Dependent variable is log(BMI).

Estimates of the effect of education and cognitive ability on BMI from OLS, single-variable MR and multivariable MR analysis of individual-level data.

All regressions also include a full set of control variables: age, gender, income and 10 genetic principal components.

Instruments are constructed from GWAS scores for education and cognitive ability. The regressions are weighted so that individuals who left school at 15 are given an 80% up-weighting. All non-European and related individuals have been excluded from the analysis. Total sample size included in all regressions: 74 309.

### Testing the instrument strength in the single-sample setting

As a measure of the strength of the instruments, we calculate the standard F-statistic for both education and cognitive ability and the Sanderson–Windmeijer partial F-statstic^33^ for the multivariable MR analysis. As all F-statistics are much larger than the rule-of-thumb cut-off of 10, we are reassured that the instruments are not individually weak. However, the partial F-statistic for both education and cognitive ability is significantly lower, showing that the power of the instruments to predict both variables simultanously is greatly reduced.

The Sargan test for invalid instruments can only be calculated for estimation models with more instruments than exposure variables. In this estimation, we have two exposures and two instruments and so it is not possible to calculate the Sargan statistic.

### Two-sample MVMR

To illustrate two-sample MVMR, we randomly divided the sample used for the individual analysis into three equally sized groups. For each SNP used in the polygenic score, we then calculated its effect on log(BMI), education and cognitive ability using different parts of the sample. The results were then used to conduct a two-sample MVMR analysis. The results are given in Supplementary Table 4, available as Supplementary data at *IJE* online. They show that increased education has a direct effect that decreases BMI and cognitive ability has no direct effect on BMI. The results are in line with those obtained from the individual-level analysis.

### Testing instrument strength in the two-sample setting

To test for weak instruments in this analysis, we have calculated the weak-instrument Q statistics for education and cognitive ability. The $Q_{edu}$ statistic for education is 1724.4. The $Q_{cog}$ statistic for cognitive ability is 1488.8. The critical value for a $\chi^{2}$ distribution with 88 degrees of freedom at the 5% level is 110.9. Therefore, we reject the null hypothesis that the SNPs do not explain any of the variation in the exposures education and cognitive ability in this two-sample analysis and can conclude that these SNPs can predict both education and cognitive ability in the data.

### Testing for pleiotropy in the two-sample setting

To illustrate the two tests for pleiotropy discussed earlier, we report the $Q_{A}$ statistic for MVMR. The value of $Q_{A}$ for this regression is 129.5. The critical value for a $\chi^{2}$ distribution with 87 degrees of freedom is 109.77. Therefore, the null hypothesis that there is no heterogeneity is rejected for this value of $Q_{A}$.

### Multivariable MR Egger regression

An alternative procedure that has been recently proposed to adjust for pleiotropy beyond that explainable by genetically predictable exposures (e.g. $X_{1}$ and $X_{2}$) is a multivariable MR Egger regression.^46^ This is a natural extension of the original MR Egger approach^1^ and is calculated by fitting the two-sample MVMR model with a constant included:

$${\hat{\Gamma }}_{j}=\beta_{0}+\beta_{1}{\hat{\pi }}_{1j}+\beta_{2}{\hat{\pi }}_{2j}+U_{Yj}+v_{Yj}$$

If the constant is different from zero, this suggests that additional pleiotropy is meaningfully biasing the analysis. However, a generalization of the InSIDE assumption is required in order for it to deliver unbiased causal estimates. These are described in detail elsewhere.^1^

The two-sample results were used to fit multivariable MR Egger regression, the results of which are given in Supplementary Table 5, available as Supplementary data at *IJE* online. Its intercept parameter is estimated to be small, and consequently the estimated effects of the exposures do not differ from those in the two-sample MVMR estimation. This supports the suggestion that the SNPs do not exert a direct effect on BMI apart from through education or cognitive ability. As MR Egger is dependent on the orientation of the SNP–exposure associations, we repeated this analysis with the associations orientated so that the SNP education associations where all positive and then with the SNP cognitive ability associations all positive. These changes had no substantive effect on the results obtained.

**Figure 7.**
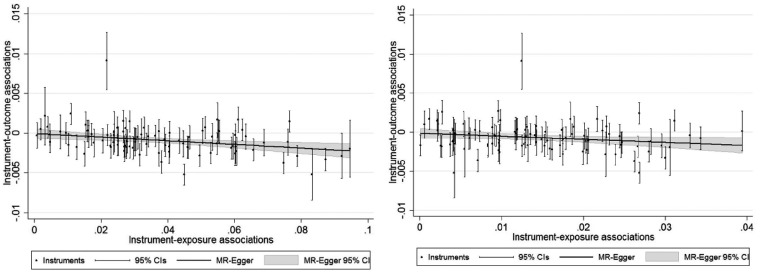
Left: MR Egger plot for the association between educational attainment and BMI. Right: MR Egger plot for the association between cognitive ability and BMI. All SNPs that affect either education or cognitive ability are included.

The difference between the Q statistic and multivariable MR Egger estimation suggests an inconsistency between these two tests, although this may have arisen due to a high level of variation in the effect of the SNPs on each exposure leading to a higher Q statistic. This is supported by Figure 7A and B, which give individual MR plots for each exposure and show that there is a large amount of variation of the SNPs on each of the exposures. Repeating this analysis with the outlying SNP excluded makes no substantive difference to the results obtained.

The MVMR Egger analysis was repeated using the effect of each SNP on education, cognitive ability and BMI taken from GWAS estimates.^44^^,^^45^^,^^47^ The magnitude of the estimated effects differ in this analysis, as the outcome variable is BMI rather than the natural log of BMI, although these results also show no pleiotropic effect of the SNPs on the outcome and a negative effect of higher education on BMI. Results from this analysis are given in Supplementary Table 5, available as Supplementary data at *IJE* online.

## Discussion

In this paper, we have attempted to explain the principled application and interpretation of instrumental variable analysis to the epidemiological setting with multiple exposures. We first focused on the individual data setting, for which it is possible to borrow well-established methods (and related software) from the econometrics literature. We then considered the two-sample setting and built upon previous research in this area by developing new tests for assessing the validity and relevance of the genetic instruments. In particular, we propose two new tests:

- modified Q statistics (in our case $Q_{x1\;}$ and $Q_{x2}$) for instrument relevance that detect ‘good’ heterogeneity if a set of SNPs can jointly and reliably predict all intermediate exposures of interest;
- a modified Q statistic, $Q_{A}$ for instrument validity that detects ‘bad’ heterogeneity if a set of SNPs contains invalid instruments.

We finally illustrated the application of MVMR using individual- and summary-level data to estimate the effect of education and cognitive ability on BMI. The results from this analysis show that increasing education leads to lower BMI and the size of this effect increases when cognitive ability is controlled for. Comparing the single-exposure MR analysis results (with all SNPs that affect educational attainment excluded) with the MVMR results for cognitive ability shows a large change in the size and direction of the effect. This result suggests that education is a mediator of the relationship between cognitive ability and BMI and any direct effect of cognitive ability on BMI is minimal.

The methods we describe can be used to estimate the effect of multiple related exposures on an outcome using either individual-level or summary-level data. Although we have focused on the case of two exposures for ease of explanation, all of these methods can be easily applied to scenarios with three or more exposures. An advantage of MVMR analysis is that SNPs that are thought to potentially affect multiple exposure variables, or where it is not clear exactly which exposure they affect, can be included when estimating the effects of the exposures on the outcome. This makes MVMR particularly useful when the exposures are closely related or one (or more) is thought to be a potential pleiotropic pathway from the SNPs to the outcome. MVMR will also produce consistent estimates when there is measurement error in any of the exposure variables and therefore is a useful method of analysis when multiple exposure variables are thought to be subject to measurement error.

As with all MR analysis, it is important to ensure that the IV assumptions are satisfied. Here, we explain how the IV assumptions apply to a MVMR analysis. We describe existing tests that can be used to test the assumptions in individual-level data and propose tests that can be used with two-sample summary-level data. These new tests are a key strength of this work, as MVMR cannot be effectively used as part of the tools a researcher has available for analysis unless the potential pitfalls of the analysis are well understood. Our applied results highlight the importance of considering the IV assumptions in the context of the particular analysis being conducted as, even when the instruments appear to be very strong for each of the exposures individually, this does not guarantee that they will be equally as strong for the exposures when estimated jointly in a MVMR model. For example, the F-statistics decrease from 195 and 310 to 36 and 37 for educational attainment and cognitive ability, respectively.

A practical limitation of the new tests we develop for two-sample summary data MVMR is the reliance on knowledge of the covariance between the effect of the SNP on each exposure. These results are not available in conventional GWAS results, and it would be infeasible to calculate them in advance for every possible combination of exposure variable that could be included in a MVMR model. Unfortunately, our work shows that this information is strictly needed for valid inference. In order to conduct these tests in summary-level data, we therefore have to make a choice about how to treat these missing pieces of information. If the data were available, it can be directly calculated from the individual -level data for the particular MVMR study being conducted. Alternatively, it could be assumed to be zero, or set to zero by using non-overlapping GWAS studies for each exposure, as the standard error of the estimated SNP effects will not correlate across different samples. This is an important limitation of the results given here for testing the assumptions of two-sample summary data MVMR.

Another weakness of the instrument relevance test we develop is that this is a test for whether the SNPs can conditionally explain any of the variation in the exposure variables, rather than being a more usual weak-instrument test, such as the rule of thumb of F being greater than 10 for a univariable MR analysis or the Sanderson–Windmeijer conditional F-statistic for IV analysis with individual-level data. Extending this test to weak instrument is an area for future work.

## Supplementary Material

dyy262_Supplementary_Tables
