# Corrigendum to: An examination of multivariable Mendelian randomization in the single-sample and two-sample summary data settings

**DOI:** 10.1093/ije/dyaa101

**Published:** 2020-06-12

**Authors:** Eleanor Sanderson, George Davey Smith, Frank Windmeijer, Jack Bowden

The caption submitted for Figure 1 was the same as the caption for Figure 2, and incorrect.

The correct caption for Figure 1 is shown below.

**Figure 1.**
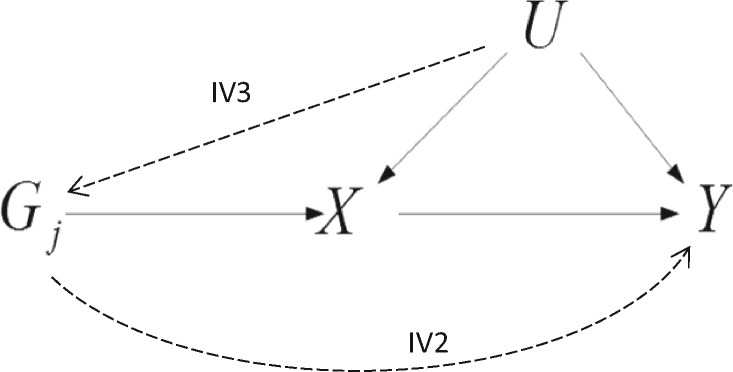
Hypothesised relationship between genetic variant *G_j_*, modifiable exposure *X* and outcome *Y* in the presence of an unobserved confounder, denoted by *U*. The line from *G_j_* to *X* represents instrumental variable assumption IV1. Dashed lines represent potential violations of the instrumental variable assumptions IV2 and IV3.

## Notes

First published online 10 December 2018, *Int J Epidemiol* 2019;48:713–27. doi: https://doi.org/10.1093/ije/dyy262

