# Supplementary material for: An examination of multivariable Mendelian randomization in the single-sample and two-sample summary data settings
Source: Int J Epidemiol. 2018 Dec 10;48(3):713–27. doi: 10.1093/ije/dyy262 (PMC6734942; doi:10.1093/ije/dyy262)
Supplement: dyy262_Supplementary_Tables [file dyy262_supplementary_tables.docx]

***Table S.1 – Methods of estimation used in the simulations.***

| **OLS estimation** | | | |
| --- | --- | --- | --- |
| Single variable regression | $y=\alpha+\beta_{1}x_{1}$ | $y=\alpha+\beta_{2}x_{2}$ |  |
| Multivariable regression | $y=\alpha+\beta_{1}x_{1}+ \beta_{2}x_{2}$ | |  |
| **Single sample methods of estimation** | | | |
| 1. Single variable MR | $y=\alpha+ \beta_{1} \hat{x}_{1}+u$  $x_{1}=\gamma_{1}+ \pi_{1}G+v_{1}$ | $y=\alpha+ \beta_{2} \hat{x}_{2}+u$  $x_{2}=\gamma_{1}+ \pi_{2}G+v_{2}$ | Individual single variable Mendelian Randomisation for each exposure variable, each predicted using all of the genetic variants available. |
| 1. Multivariable MR | $y=\alpha+ \beta_{1} \hat{x}_{1}+ \beta_{2} \hat{x}_{2}+u$  $x_{1}=\gamma_{1}+ \pi_{1}G+v_{1}$  $x_{2}=\gamma_{2}+ \pi_{2}G+v_{2}$ | | Multivariable Mendelian Randomisation with both the exposures included and predicted using all of the genetic variants available. |
| 1. Single variable MR Using a subset of the SNPs available. | $y=\alpha+ \beta_{1} \hat{x}_{1}+u$  $x_{1}=\gamma_{1}+ \pi_{1}G_{1}+v_{1}$ | $y=\alpha+ \beta_{1} \hat{x}_{1}+u$  $x_{1}=\gamma_{1}+ \pi_{1}G_{2}+v_{1}$ | Individual single variable Mendelian Randomisation for each exposure variable, each predicted using only the SNPs known to be associated with only that exposure. |
| **Two-sample methods of estimation** | | | |
| 1. Single variable MR | $\hat{\Gamma}= \beta_{1} \hat{\pi}_{1}+u$  $y= \Gamma_{j}G_{j}+ \epsilon_{j}$  $x_{1}= \pi_{1,j}G_{j}+v_{1,j}$ | $\hat{\Gamma}= \beta_{2} \hat{\pi}_{2}+u$  $y= \Gamma_{j}G_{j}+ \epsilon_{j}$  $x_{2}= \pi_{2,j}G_{j}+v_{2,j}$ | Individual single variable Mendelian Randomisation for each exposure variable, each predicted using all of the genetic variants available. |
| 1. Multivariable MR | $\hat{\Gamma}= \beta_{1} \hat{\pi}_{1}+ \beta_{2} \hat{\pi}_{2}+u$  $y= \Gamma_{j}G_{j}+ \epsilon_{j}$  $x_{1}= \pi_{1,j}G_{j}+v_{1,j}$  $x_{2}= \pi_{2,j}G_{j}+v_{2,j}$ | | Multivariable Mendelian Randomisation with both exposure variables included and predicted using all of the genetic variants available. |
| 1. Single variable MR Using a subset of the SNPs available. | $\hat{\Gamma}= \beta_{1} \hat{\pi}_{1}+u$  $y= \Gamma_{j}G_{1,j}+ \epsilon_{j}$  $x_{1}= \pi_{1,j}G_{1,j}+v_{1,j}$ | $\hat{\Gamma}= \beta_{2} \hat{\pi}_{2}+u$  $y= \Gamma_{j}G_{2,j}+ \epsilon_{j}$  $x_{2}= \pi_{2,j}G_{2,j}+v_{2,j}$ | Individual single variable Mendelian Randomisation for each exposure variable, each predicted using only the SNPs known to be associated with only that exposure. |

***Table S.2 –Simulation results***

***S.2.a Confounding Setup***

| **Method of Estimation** | ${\hat{\boldsymbol{\beta}}}_{\boldsymbol{1}}$ | **std. error** ${\hat{\boldsymbol{\beta}}}_{\boldsymbol{1}}$ | ${\hat{\boldsymbol{\beta}}}_{\boldsymbol{2}}$ | **std. error** ${\hat{\boldsymbol{\beta}}}_{\boldsymbol{2}}$ |
| --- | --- | --- | --- | --- |
| *OLS estimation* |  |  |  |  |
| Single variable | 1.88 | 0.046 | 2.31 | 0.067 |
| Multivariable | 1.25 | 0.032 | 1.13 | 0.044 |
| *Single Sample MR estimation* |  |  |  |  |
| 1. Univariate MR | 1.53 | 0.132 | 1.84 | 0.132 |
| 1. MVMR | 1.00 | 0.017 | 1.00 | 0.022 |
| 1. Univariate MR – subset of SNPS | 1.01 | 0.044 | 1.51 | 0.045 |
| *Two Sample MR estimation* |  |  |  |  |
| 1. Univariate MR | 1.52 | 0.141 | 1.82 | 0.144 |
| 1. MVMR | 0.99 | 0.072 | 0.99 | 0.091 |
| 1. Univariate MR – subset of SNPS | 0.99 | 0.088 | 1.49 | 0.092 |

Data is set up as given in Fig. 4 scenario 1. $\beta_{1}=1$, $\beta_{2}=1$, $\gamma_{1}=0.5$.

The total and direct effect of $X_{1}$ = 1, total effect of $X_{2}=1.5$, direct effect of $X_{2}=1$.

n= 20,000. 1000 repetitions.

***S.2.b Collider Setup***

| **Method of Estimation** | ${\hat{\boldsymbol{\beta}}}_{\boldsymbol{1}}$ | **std. error** ${\hat{\boldsymbol{\beta}}}_{\boldsymbol{1}}$ | ${\hat{\boldsymbol{\beta}}}_{\boldsymbol{2}}$ | **std. error** ${\hat{\boldsymbol{\beta}}}_{\boldsymbol{2}}$ |
| --- | --- | --- | --- | --- |
| *OLS estimation* |  |  |  |  |
| Single variable | 1.36 | 0.031 | 0.71 | 0.019 |
| Multivariable | 0.65 | 0.054 | 0.43 | 0.032 |
| *Single Sample MR estimation* |  |  |  |  |
| 1. Univariate MR | 1.00 | 0.016 | 0.51 | 0.084 |
| 1. MVMR | 0.99 | 0.028 | 0.01 | 0.018 |
| 1. Univariate MR – subset of SNPS | 1.00 | 0.025 | 0.02 | 0.045 |
| *Two Sample MR estimation* |  |  |  |  |
| 1. Univariate MR | 0.99 | 0.038 | 0.50 | 0.087 |
| 1. MVMR | 1.00 | 0. 066 | 0.00 | 0.040 |
| 1. Univariate MR – subset of SNPS | 0.99 | 0.055 | 0.00 | 0.045 |

Data is set up as given in Fig. 4 scenario 2. $\beta_{1}=1$, $\beta_{2}=0$,$\alpha_{1}=0.5$.

The total and direct effect of $X_{1}$ = 1, total and direct effect of $X_{2}=0$.

n= 20,000. 1000 repetitions.

***S.2.c Pleiotropic Setup***

| **Method of Estimation** | ${\hat{\boldsymbol{\beta}}}_{\boldsymbol{1}}$ | **std. error** ${\hat{\boldsymbol{\beta}}}_{\boldsymbol{1}}$ | ${\hat{\boldsymbol{\beta}}}_{\boldsymbol{2}}$ | **std. error** ${\hat{\boldsymbol{\beta}}}_{\boldsymbol{2}}$ |
| --- | --- | --- | --- | --- |
| *OLS estimation* |  |  |  |  |
| Single variable | 1.81 | 0.063 | 1.81 | 0.067 |
| Multivariable | 1.25 | 0.032 | 1.25 | 0.033 |
| *Single Sample MR estimation* |  |  |  |  |
| 1. Univariate MR | 1.34 | 0.129 | 1.34 | 0.133 |
| 1. MVMR | 1.00 | 0.018 | 1.00 | 0.018 |
| 1. Univariate MR – subset of SNPS | 1.00 | 0.045 | 1.01 | 0.045 |
| *Two Sample MR estimation* |  |  |  |  |
| 1. Univariate MR | 1.32 | 0.137 | 1.33 | 0.138 |
| 1. MVMR | 0.99 | 0.060 | 0.99 | 0.060 |
| 1. Univariate MR – subset of SNPS | 0.99 | 0.072 | 0.99 | 0.073 |

Data is set up as given in Fig. 4 scenario 3. $\beta_{1}=1$, $\beta_{2}=0$,$\alpha_{1}=0$.

The total and direct effect of $X_{1}$ = 1, total and direct effect of $X_{2}=1$.

n= 20,000. 1000 repetitions.

***S.2.d Mediation Setup***

| **Method of Estimation** | ${\hat{\boldsymbol{\beta}}}_{\boldsymbol{1}}$ | **std. error** ${\hat{\boldsymbol{\beta}}}_{\boldsymbol{1}}$ | ${\hat{\boldsymbol{\beta}}}_{\boldsymbol{2}}$ | **std. error** ${\hat{\boldsymbol{\beta}}}_{\boldsymbol{2}}$ |
| --- | --- | --- | --- | --- |
| *OLS estimation* |  |  |  |  |
| Single variable | 2.31 | 0.063 | 1.88 | 0.048 |
| Multivariable | 1.12 | 0.043 | 1.25 | 0.033 |
| *Single Sample MR estimation* |  |  |  |  |
| 1. Univariate MR | 1.84 | 0.129 | 1.54 | 0.133 |
| 1. MVMR | 1.00 | 0.021 | 1.00 | 0.018 |
| 1. Univariate MR – subset of SNPS | 1.51 | 0.045 | 1.01 | 0.045 |
| *Two Sample MR estimation* |  |  |  |  |
| 1. Univariate MR | 1.82 | 0.142 | 1.52 | 0.142 |
| 1. MVMR | 0.99 | 0.091 | 0.99 | 0.073 |
| 1. Univariate MR – subset of SNPS | 1.49 | 0.090 | 0.99 | 0.088 |

Data is set up as given in Fig. 4 scenario 4. $\beta_{1}=1$, $\beta_{2}=0$,$\alpha_{1}=0.5$.

The total effect of $X_{1}$ = 1.5, direct effect of $X_{1}$ = 1, total and direct effect of $X_{2}=1$.

n= 20,000. 1000 repetitions.

***Table S.3 – Educational qualifications***

| **Highest Educational qualification** | **Age completed education** | **% of final sample** |
| --- | --- | --- |
| None | 15 | 12.29 |
| CSE’s/O levels/GCSEs | 16 | 27.61 |
| NVQ/HND/HNC | 18 | 6.41 |
| A levels | 18 | 12.23 |
| Other professional qualification  (e.g. Nursing/Teaching etc) | 20 | 4.98 |
| College or University degree | 21 | 36.47 |

The highest reported educational qualification and associated age for completing education

for the individuals from UK biobank included in this analysis.

***Table S.4 – Two-sample Multivariable MR estimation***

|  | Effect | Std. Error | 95% Confidence Interval | P-value |
| --- | --- | --- | --- | --- |
| *Log BMI* |  |  |  |  |
| Age completed Education | -0.022 | 0.006 | [-0.034, -0.010] | <0.001 |
| Standardised Cognitive ability score | 0.007 | 0.016 | [-0.026, 0.039] | 0.685 |

Estimates of the effect of Education and cognitive ability on Log BMI from a two-sample analysis

3 SNPs in the education GWAS which are in LD with SNPs from the cognitive ability GWAS have been excluded.

Each sample includes on third of the observations in the total sample

The effects of each SNP on log BMI, education, cognitive ability have each been calculated from one sub–sample only.

***Table S.5 - Multivariable MR Egger estimation***

|  | Effect | Std. Error | 95% Confidence Interval | P-value |
| --- | --- | --- | --- | --- |
| *Biobank data - Log BMI* |  |  |  |  |
| Age completed Education | -0.022 | 0.006 | [-0.033, -0.010] | <0.001 |
| Standardised Cognitive ability score | 0.007 | 0.016 | [-0.025, 0.039] | 0.667 |
| Constant | -0.0003 | 0.0002 | [-0.0008, 0.0001] | 0.166 |
| *Summary GWAS data – BMI* |  |  |  |  |
| Age completed Education | -0.345 | 0.098 | [-0.542, -0.146] | 0.001 |
| Standardised Cognitive ability score | 0.046 | 0.073 | [-0.102, 0.194] | 0.532 |
| Constant | -0.001 | 0.001 | [-0.004, 0.001] | 0.254 |

Multivariable MR Egger estimates for the effect of education and cognitive ability on BMI.

The first section shows the estimated effects calculated using UK biobank. the estimation has been constructed in the same way as given in Table A.4.

The second section shows the results calculated using summary GWAS data for education, cognitive ability and BMI [44, 45],[47]

***Stata code to reproduce the results for the analysis of education and cognitive ability on BMI***

This analysis uses UK biobank data with cleaning and exclusions applied as described in the paper.

*******************************

**ANALYSIS WITH INDIVIDUAL DATA

*******************************

*********

**Table 4

*********

global controls age i.income female pc1 - pc10

**multivariable MR regression

ivreg2 bmi_ln (eduyears CA_std = allele_edu allele_CA) $controls [pweight=weight], robust ffirst

gen inreg = e(sample)

keep if inreg == 1

*Single variable MR regressions

ivreg2 bmi_ln (eduyears = allele_edu) $controls [pweight=weight] if inreg == 1, robust ffirst

ivreg2 bmi_ln (CA_std = allele_CA) $controls [pweight=weight] if inreg == 1, robust ffirst

**OLS regressions

*multivariable

reg bmi_ln eduyears CA_std $controls [pweight=weight] if inreg == 1, robust

*single variable

reg bmi_ln eduyears $controls [pweight=weight] if inreg == 1, robust

reg bmi_ln CA_std $controls [pweight=weight] if inreg == 1, robust

*********************

**TWO SAMPLE ANALYSIS

*********************

**create the two sample effect sizes and standard errors from the biobank data

**these are created in this way to illustrate the analysis in a two sample setting where the samples are known to come from the same population

use "analysis data.dta", clear

global edu_snps rs10061788 rs1008078 rs1043209 rs10496091 rs11191193 rs11210860 rs112634398 rs113520408 rs17538393 rs11588857 rs11689269 rs11690172 rs11712056 rs11768238 rs12531458 ///

rs12646808 rs12671937 rs12682297 rs12772375 rs12969294 rs12987662 rs13294439 rs13402908 rs1402025 rs9878943 rs1606974 rs165633 rs16845580 rs17119973 rs17167170 rs1777827 ///

rs17824247 rs1871109 rs2245901 rs2431108 rs2456973 rs2457660 rs2568955 rs2610986 rs2615691 rs2837992 rs2964197 rs2992632 rs301800 rs3101246 rs324886 rs34072092 rs34305371 ///

rs35761247 rs4493682 rs4500960 rs4851251 rs4863692 rs55830725 rs55943044 rs56231335 rs572016 rs61160187 rs62259535 rs62263923 rs62379838 rs6739979 rs6799130 rs7131944 rs7306755 ///

rs76076331 rs7767938 rs7854982 rs7945718 rs7955289 rs8008779 rs895606 rs1487445 rs9537821

global Cognition_snps rs10191758 rs10236197 rs10733787 rs11138902 rs12744310 rs12928404 rs13010010 rs1626122 rs16954078 rs2251499 rs2490272 rs2743462 rs41352752 rs4728302 ///

rs6746731 rs6779302 rs7646501 rs78164635 /*Note these IQ snps are based on the Sniekers et al. Intelligence in UK Biobank paper*/

*create the two sample results to use to illustrate the analysis

gen part = _n <= _N/3

replace part = 2 if _n > _N/3 & _n <= (2*_N)/3

replace part = 3 if _n > (2*_N)/3

reg eduyears rs10061788 if part == 2

regsave using results, addlabel(depvar, edupart) replace

foreach x in $edu_snps $Cognition_snps {

reg bmi_ln `x' if part == 1

regsave using results, addlabel(depvar, bmi_ln) append

reg eduyears `x' if part == 2

regsave using results, addlabel(depvar, eduyears) append

reg CA_std `x' if part == 3

regsave using results, addlabel(depvar, CAstd) append

}

use results, clear

duplicates drop

drop if var == "_cons"

reshape wide coef stderr N r2 , i(var) j(depvar) string

rename var SNP

**drop the SNPs in education that are in LD with one of the cognition SNPs

drop if SNP == "rs12987662" | SNP == "rs17824247" | SNP == "rs17167170"

*********

*Analysis

*********

*two-sample MVMR with simple weights

reg coefbmi_ln coefCAstd coefeduyears [aweight = 1/(stderrbmi_ln^2)], noc

/*

Pleiotropy test

Q statistic for two sample MVMR with adjusted weights

This calculates the test statistic given in equation 13.

*/

*creating the weights

predict fitted, xb

gen double weight = stderrbmi_ln^2 + (_b[coefCAstd]^2)*(stderrIQstd^2) + (_b[coefeduyears]^2)*(stderreduyears^2)

*create the test statistic

egen Q = sum(((coefbmi_ln - fitted)^2)/weight)

tab Q

**********

*Table A.4

**********

*two-sample MVMR with updated weights

reg coefbmi_ln coefCAstd coefeduyears [aweight = 1/(weight)], noc

/*

Test of instrument strength

Q statistic for two sample MVMR to test for underidentification in the first stage

This calculates the test statistic given in equation 12.

*/

*estimate delta for the regressions of education on cognitive ability and cognitive ability on education

reg coefeduyears coefCAstd, noc

gen deltaed = _b[coefCAstd]

reg coefCAstd coefeduyears, noc

gen deltaCA = _b[coefeduyears]

*calculate the Q statistics for education and cognitive ability

egen Qed = sum(((coefeduyears - deltaed*coefCAstd)^2)/(scvar_edu + (deltaed^2)*scvar_CA))

summ Qed

egen QCA = sum(((coefCAstd - deltaCA*coefeduyears)^2)/(scvar_CA + (deltaCA^2)*scvar_edu))

summ QCA

*********************

**MR Egger estimation

*********************

/*

It is not clear how to adjust the coefficients with multiple exposures so estimate the MR Egger regression unadjusted, adjusted for education and adjusted for Cognitive ability separately.

(Rees, J.M., Wood, A.M. and Burgess, S., 2017. Statistics in medicine.)

*/

*adjust according to education

gen coefedu_adj = abs(coefeduyears)

gen bmi_adje = coeflnbmi

replace bmi_adje = -bmi_adje if coefeduyears <0

gen ca_adje = coefCAstd

replace ca_adje = -ca_adje if coefeduyears <0

*adjust according to IQ

gen coefca_adj = abs(coefCAstd)

gen bmi_adjca = coeflnbmi

replace bmi_adjca = -bmi_adjca if coefCAstd <0

gen edu_adjca = coefeduyears

replace edu_adjca = -edu_adjca if coefCAstd <0

**un adjusted

reg coeflnbmi coefCAstd coefeduyears [aweight = 1/(stderrlnbmi^2)]

**adjusted for education

reg bmi_adje CA_adje coefedu_adj [aweight = 1/(stderrlnbmi^2)]

**adjusted for cognitive ability

reg bmi_adjca coefCA_adj edu_adjca [aweight = 1/(stderrlnbmi^2)]

*create the MR Egger plots for education and cognitive ability separately.

*Figure 7a

mreggerplot coeflnbmi stderrlnbmi coefeduyears stderreduyears

*Figure 7b

mreggerplot coeflnbmi stderrlnbmi coefCAstd stderrCAstd

****************************

**MVMR EGGER using GWAS data

****************************

/*

This analysis uses data from previously published GWAS to conduct a two-sample summary data multivariable MR Egger - given in table A.5.

GWAS results are from:

Okbay, A., et al. Nature, 2016.

Sniekers, S., et al. Nature Genetics, 2017.

Speliotes, E.K., et al. Nat Genetics, 2010.

*/

use "GWASdata.dta", clear

**SNPs in education that are in LD with one of the cognition SNPs

drop if SNP == "rs12987662" | SNP == "rs17824247" | SNP == "rs17167170"

reg bmibeta CAbeta edubeta [weight = bmise^-2]
